# Spatial heterogeneity and risk factors for stunting among children under age five in Ethiopia: A Bayesian geo-statistical model

**DOI:** 10.1371/journal.pone.0170785

**Published:** 2017-02-07

**Authors:** Seifu Hagos, Damen Hailemariam, Tasew WoldeHanna, Bernt Lindtjørn

**Affiliations:** 1 Department of Reproductive Health and Health Service Management, School of Public Health, College of Health Sciences, Addis Ababa University, Addis Ababa, Ethiopia; 2 Center for International Health, University of Bergen, Bergen, Norway; 3 Department of Economics, College of Business and economics, Addis Ababa University, Addis Ababa, Ethiopia; Brighton and Sussex Medical School, UNITED KINGDOM

## Abstract

**Background:**

Understanding the spatial distribution of stunting and underlying factors operating at meso-scale is of paramount importance for intervention designing and implementations. Yet, little is known about the spatial distribution of stunting and some discrepancies are documented on the relative importance of reported risk factors. Therefore, the present study aims at exploring the spatial distribution of stunting at meso- (district) scale, and evaluates the effect of spatial dependency on the identification of risk factors and their relative contribution to the occurrence of stunting and severe stunting in a rural area of Ethiopia.

**Methods:**

A community based cross sectional study was conducted to measure the occurrence of stunting and severe stunting among children aged 0–59 months. Additionally, we collected relevant information on anthropometric measures, dietary habits, parent and child-related demographic and socio-economic status. Latitude and longitude of surveyed households were also recorded. Local Anselin Moran's *I* was calculated to investigate the spatial variation of stunting prevalence and identify potential local pockets (hotspots) of high prevalence. Finally, we employed a Bayesian geo-statistical model, which accounted for spatial dependency structure in the data, to identify potential risk factors for stunting in the study area.

**Results:**

Overall, the prevalence of stunting and severe stunting in the district was 43.7% [95%CI: 40.9, 46.4] and 21.3% [95%CI: 19.5, 23.3] respectively. We identified statistically significant clusters of high prevalence of stunting (hotspots) in the eastern part of the district and clusters of low prevalence (cold spots) in the western. We found out that the inclusion of spatial structure of the data into the Bayesian model has shown to improve the fit for stunting model. The Bayesian geo-statistical model indicated that the risk of stunting increased as the child’s age increased (OR 4.74; 95% Bayesian credible interval [BCI]:3.35–6.58) and among boys (OR 1.28; 95%BCI; 1.12–1.45). However, maternal education and household food security were found to be protective against stunting and severe stunting.

**Conclusion:**

Stunting prevalence may vary across space at different scale. For this, it's important that nutrition studies and, more importantly, control interventions take into account this spatial heterogeneity in the distribution of nutritional deficits and their underlying associated factors. The findings of this study also indicated that interventions integrating household food insecurity in nutrition programs in the district might help to avert the burden of stunting.

## Introduction

Child undernutrition, including macronutrient and micronutrient deficiencies, contributed around 45% of child deaths in 2011 [1]. Stunting, a measure of chronic undernutrition, is the most prevalent form of child undernutrition in developing countries. Thus, in 2011, about 165 million stunted children were estimated in developing countries[2]. Globally, the prevalence of stunting among children under age five has decreased. At regional level, as compared to other regions, very little decline in the prevalence of stunting is documented in Africa[2]. In 2012, the WHO sets a global target to reduce the number of stunted children by 40% from the baseline 171 million in 2010 to 100 million by 2025[2].

Stunting, defined as height-for-age (HAZ) z- scores 2 standard deviations (SD) below WHO 2005 median, is a serious public health problem and its consequence goes beyond the childhood period. Evidences suggest that stunting has both short and long term consequences such impaired health, lower school and educational performance, lower economic productivity and loss of human capital and household income [3,4], and increased risk of cardiovascular diseases in adulthood [5].

Ethiopia has given a priority to reduce stunting as established in its national nutrition strategy and plan. This plan of action aims to accelerate the reduction of stunting and focuses on the first 1000 days, children under two years and pregnant and lactating women [6]. Despite the country has documented a decline in the prevalence of stunting from 58% to 40% between 2000 and 2014 [7–10], prevalence rates still remain high. Moreover, the progress in reduction of stunting among the regions is uneven across the country. Based on the WHO recommended criteria for assessing the severity of stunting at population level, 5 out of the nine regions in the country are classified under very high severity (>40% prevalence) while the remaining 4 were classified in the medium to high severity (prevalence range of 20–40%).

The factors influencing stunting among children 0–59 months are multiple. The WHO conceptual framework for stunting (2013) identified household and family factors, complementary feeding and breastfeeding practices and infections as the most plausible causes of stunting [11]. Contextual factors at community and societal level such as beliefs, norms, and livelihoods influence the proximate causes of stunting. Many studies corroborated in reporting significant association with maternal education, household wealth, maternal age, dietary diversity, diseases and infections. Dietary diversity and recommended complementary feeding practices were found to be significantly associated with reducing the odds of stunting in younger children [12,13]. Infectious diseases arising from respiratory illnesses [14], malaria [15] and diarrhoea [14–16] during the period of growth and development (6–59 months) are also important proximal determinants of growth faltering [17,18]. At the individual level, stunting prevalence appears to be more prevalent in boys than girls and also in older age groups [1].

The underlying factors for child undernutrition also include household and family related factors such as food security, child care, household environment (access to water and sanitation) and lack of basic health services [1,11,18,19]. Household economic inequalities and its relation to child undernutrition (stunting) is a controversial issue. Some studies have shown the potential of improved household income in improving access and the consumption of goods and services, which in turn improves undernutrition [1,20–22]. In contrast others have reported a weak relation between income growth and undernutrition at the household level [23–25]. The mother’s education, which is a modifiable household factor, is a key predictor of linear growth and stunting [11,26–30]. A better maternal educational status might improve child caring practices related to stunting, or could help to understand and respond to nutrition-based educational messages, as well as helping to adopt optimal complementary feeding and household dietary behaviours [30].

In summary we have noted a significant variation among studies on the relative importance of factors associated with stunting and undernutrition. We believe that the variation in findings arises partly from the underlying social environment (contexts) upon which the different risk factors for stunting operate. In addition to this, quite a few of these studies accounted for the spatial dependence structures in the data when analyzing the risk factors for stunting [31–33]. Failure to account for spatial dependency, when existing, could result in spurious significant association. Hence it is important to understand contextual factors influencing stunting in Ethiopia. Therefore, in the present work we aimed at identifying risk factors for stunting and severe stunting in southern Ethiopia and explored their spatial distribution at district (meso-scale) level. Additionally, we investigated the effect of spatial dependency on the identification of risk factors and their relative contribution to the occurrence of stunting and severe stunting. The findings from this study will provide new insights on the spatial epidemiology of stunting and more practically will help local health services tackle more efficiently the underlying factors behind the occurrence of stunting in the study area and other similar contexts.

## Materials and methods

### Study setting

We conducted this study in Meskane Mareko District (38.45763 E, 8.042144 N), which is located around 130 km south of Addis Ababa (capital city of Ethiopia). The district is located in Guraghe Zone, in the Southern part of the country. According to the district Office report, the district had an estimated population size of 199,771 and 38, 933 households. Seven health centers, 40 health posts and two hospitals were serving the residents in the district at the time of the study.

### Study design and period

This study used a cross sectional design. The data collection was conducted between December 2013 and April 2014.

### Sample size and sampling procedure

The data for this work was extracted from a major study intended to examine the spatial distribution of the three forms of undernutrition; wasting, underweight and stunting at different geographic scale in the district. We calculated sample sizes for multiple objectives of the research project using a formula for estimating a single population proportion. Three sample sizes were determined taking the expected prevalence of severe wasting, severe underweight and severe stunting and using the following parameters; 80% power and 95% confidence level, design effect of 1.5 and 1.0% margin of error. By comparing the sample sizes calculated for the three objectives, the largest sample size was obtained for the expected prevalence of severe wasting [34]. Using the expected prevalence of severe wasting, the calculated sample size was 1,567 children in the age group of 0–59 months. With an expectation of approximately 10% for non-response rate, a minimum of 1,723 children under age five were required. This sample size was chosen since it was adequate to answer the other objectives of the study.

The district had a total of 40 *kebeles* (*Kebele is the smallest administrative unit in Ethiopia*). A *kebele* is further divided into villages. The sample size (1,723 children) was allocated to 34 kebeles proportional to the number of households. For each *kebele*, we randomly selected one or two villages depending on the number of villages per *kebele*. For *Kebeles* with 3–6 villages, we randomly selected one village while for *Kebeles* with 7–9 villages; we randomly selected two villages from each *Kebele*. This gave us a total of 45 villages. The sample size (1,723 children) was divided among the 45 villages proportionate to the number of households.

We obtained the list of household’s names and profile from the Health Extension Workers’ (HEWs) family registry forms. Households were eligible for the study if their occupants have children under the age of five. Households (secondary sampling unit) were selected using simple random sampling method. We visited all households with support of HEWs, local guides and local supervisors. All children aged 0–59 months found in sampled houses were included in the study.

Few days before this survey, we completed a census on selected six kebeles (24 villages) found in the district to evaluate the local scale (within kebeles) spatial distribution and clustering of wasting, underweight and stunting[35]. We used the same research tool and research assistants with that of this survey. We collected data from a total of 2,371 children under the age of five. The study team subsequently decided to include this sample to the present analytic sample in order to improve and guarantee the representativeness of the outcome. Potential bias on different sampling design was minimized by giving more weight to children selected randomly at the main survey. This paper is hence based on a total 4, 094 children under age five who lived in 40 *kebeles* in the district.

### Data collection

#### Anthropometry data

Anthropometric data (weight, height/length) were collected from children aged 0–59 months. A digital weighing scale (Coline brand) with weighting capacity of 150kg and designed in 100gram graduation was used to measure weight of children. For some of the children, we used tare weighing. Locally constructed Stadiometer was used to measure height of children whereas length boards were used to measure length of children. Length/height measurements were taken with a precision of 0.1cm. Due to the lack of accurate record of date of birth, we used local calendar of events to estimate children and mother’s month and year of birth.

Twenty enumerators were trained on anthropometric measurement, geo referencing visited households and interviewing skills. We evaluated the performance of the enumerators through conducting a measurement standardization exercise on five children. We paired the enumerators to form ten teams. Each team measured the height of the five children in two rounds. These two rounds of measurements were used to calculate the intra-pair Technical error measurement (TEM) and inter-pair TEM. The intra Pair TEM was used to evaluate variation in measurement within pairs of data collectors whereas the inter-pair TEM was used to evaluate the variation in measurement between pairs of data collectors. We calculated the coefficient of reliability (R) that is used to estimate the proportion of total variance that cannot be explained by measurement error. This standardization exercise was repeated until the coefficient of reliability was 86%. The methods employed in constructing the coefficient of reliability and detailed report are provided elsewhere[35].

We administered a questionnaire to mothers or guardian of children to collect a range of factors that can be associated with stunting. The questionnaire included items on household and family factors concerning home environment, sanitation, food and water safety, household food access, dietary diversity, history of morbidity (diarrheal diseases and fever) and child/parent socio demographic characteristics [11]. Geographic location of selected households and their elevation were collected using a hand held Global Positioning System (GPS) device (Garmin GPSMAP^®^).

The questionnaire included the Household food Insecurity Assessment Scale (HFIAS) for determining the household food insecurity (access) prevalence. The HFIAS was developed by Food and Nutrition Technical Assistance Project to measure the magnitude of food Insecurity at household level. The HFIAS tool is based on a set of nine questions and follow-up of frequency of occurrence questions. The HFIAS tool was validated and has shown to perform well with minor adaptations in the same setting [36].

In addition, household food intake was qualitatively evaluated through asking consumption of twelve food groups. We asked the consumption of the following 12 food groups, including (i) cereals/breads, (ii) beans, (iii)potatoes and other roots/tubers, (iv) vegetables, (v) fruits, (vi) eggs, (vii) milk and milk products, (viii) fish, (ix) meat, (x) oil, fat or butter, (xi) sugar or honey, and, (xii) coffee and tea the day and night prior of the survey.

In order to construct a relative households wealth index, a suite of several socio economic indicators were collected: land ownership, type of house and building materials, availability of fixed domestic assets (i.e. radio, television, bed, chairs and other household items), ownership of domestic animals, source of drinking and cooking water and availability and type of latrine.

### Data analysis

We used EpiData Version 3.1 for data entry and Stata 13.0 (StataCorp, College Station, TX) for cleaning and initial analysis. We revisited the questionnaire for correcting data entry errors and missing data. All variables with missing data were reported.

#### Children nutritional status

Children were considered stunted if z-scores of height-for-age (HAZ) were 2SD below the WHO 2005 median or severe stunted if z-scores of height-for-age (HAZ) were 3SD below the WHO 2005 median. The HAZ scores were calculated using WHO Antro software (v3.2.2) and according to recent WHO reference standard [37]. Estimates of stunting and severe stunting in the district were computed using the “svy” command in STATA which accounts for clustering and stratification. Weighting was applied during the analysis whereby data from the census were given less weight than data obtained from random sampling. A complex survey data analysis was employed designating the survey’s primary sampling unit (villages) and strata (agro ecology zone). The variance was adjusted using Taylor linearized variance estimation method.

#### Household food insecurity

The household food insecurity (access) prevalence was derived from the HFIAS tool. The procedure used to assign households to one of the food insecurity levels are described elsewhere [38]. Briefly, the frequencies of affirmative responses to the nine questions describing food insecurity scenarios were used to classify households. The nine scenarios are ordered in such a way to reflect an increasing severity of food insecurity.

A household is considered food secured when none of the 9 scenarios are experienced or only sporadic "concern about food" is reported (first scenario). A severely food insecured household experiences one of the last three scenarios (items 7, 8 and9): running out of food, going to bed hungry, or going a whole day and night without eating. Based on this, households were categorized into four levels of food insecurity: food secure, mildly food insecure, moderately food insecure and severely food insecure.

#### Dietary diversity

Household level dietary diversity indicator used in the analysis was generated using a set of twelve food groups eaten in the respective house during the day and night prior to the date of the survey. For households in the sample, we calculated the total number of food groups consumed. The total number of food groups consumed by households could range from 0 to 12. The total number of food groups consumed was used to classify households into lowest, medium and high dietary diversity. Households with lowest dietary diversity consumed three or less food groups. Households with medium dietary diversity consumed four or five food groups whereas households with high dietary diversity consumed six or more food groups.

#### Household wealth

Principal component analysis (PCA) was used to construct a relative household wealth index combining several socio economic indicators. A relative socio- economic status was constructed by dividing the resulting score into quintiles that indicate poorest, poor, medium, rich and richest households.

### Analysis of spatial distribution of stunting and severe stunting

**Fig 1:**
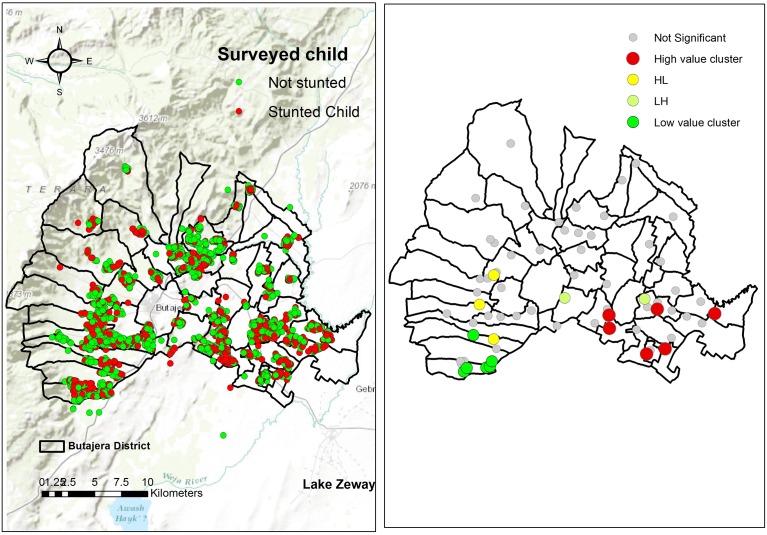
Geographic distribution of stunting, (right) hot spots of stunting (high value and low value clusters) among children under the age of 5 years in the Meskane Mareko District, Ethiopia, 2014.

We used semivariogram analysis to determine if there exists a spatial autocorrelation in the data and to evaluate whether there is a spatial trend on how stunting is spatially distributed considering both distance between observations and direction of this spatial trend. Semivariogram revealed the presence of autocorrelation by plotting the semivariance of the pairs of observations that are separated by the same distance; thus describing how similar observations are at different separation distances[39]. When the separation distance between the observations increase, the semivariance is expected to increases since observations which are closer to one another are expected to be more similar than those which are distant. Leveling out of the semivariogram indicates lack of spatial autocorrelation. The semivariogram is described by three parameters; range (maximum distance at which one could find spatial dependency), sill (the maximum semivariance value or the value where the semivariogram level out) and nugget (represents the micro-scale variation or measurement error). We used raw data to construct isotropic empirical semivariogram (employing exponential model) in R version 3.0.2. using the geoR package (Fig 1).

Additionally, spatial pattern of the occurrence of stunting was explored at district level (between communities). Thus, Anselin local Moran's *I* was used to test whether stunting were distributed randomly over space, and if not, to identify significant spatial clusters. The implementation of Anselin Local Moran's I was conducted using the tool available at the Spatial Statistic toolbox of ArcGIS version10.0 (Environmental Systems Research Institute Inc., Redlands CA, USA)[39].

We prepared an attribute table containing information for each village such as the village identification (ID), the proportion of children stunted (population file), and village’s coordinates. We assumed that spatial autocorrelation for stunting declined with the distance and therefore a spatial weight matrix conceptualizing the spatial relationship between communities was generated using an inverse distance approach.

Standardization of spatial weights was applied so that all weights summed to unity within a group of neighbours. Furthermore, we checked out for normality of the stunting prevalence data using histogram plots and Shapiro-Wilk test for normality as spatial association can be biased when data are not normally distributed.

### Variable selection

The main outcomes of interest for this study were both moderate and severe forms of stunting in children under age five. The potential explanatory (exposure) variables considered were child’s age, sex, morbidity, and place of delivery, parent or guardian’s education level, age, ethnicity, and religion, altitude, level of household food insecurity and diversity, relative economic status, household head’s gender, and husband’s occupation.

Descriptive analysis was done on explanatory variables using frequency distribution, tables, and summary statistics. The variables were checked for normality using histogram plots. Due to the problem of heaping, we transformed reported child age into six categories (<6, 6–11, 12–13, 24–35, 36–47 and 48–59 months). We checked for collinearity among explanatory variables by calculating the variance inflation factor (VIF).

A univariable multilevel analysis based on mixed effect logistic regression was used to identify factors that could potentially be associated with the occurrence of stunting and severe stunting. This univariable analysis was conducted in Stata 11.0 (StataCorp College Station, TX). Two level logistic models with a random intercept for *Kebeles* and a random intercept for villages were specified. In the final model for stunting and severe stunting, child’s age, sex, morbidity status, and place of delivery, parent or guardian education level, ethnicity, and marital status, altitude, level of household food insecurity, and relative economic status were statistically significant at 20% significance level (at P-value <0.2) and considered as potential explanatory variables for Bayesian modeling.

### Spatial and non-spatial Bayesian modeling

Bayesian logistic regression model was undertaken using WinBUGS version 1.4.3 (MRC Biostatistics Unit, Cambridge and Imperial College London, UK). Separate models were fitted for stunting and severe stunting with the variables identified above, including a Bayesian logistic regression model with a spatial dependence structure of the data and a Bayesian logistic regression model without of a spatial structure. The rationales for excluding or including the spatial dependence component were to evaluate the effect of including spatial structure on the performance of the model and also to determine the effect on the statistical significances of the odds ratios of the explanatory variables. To this end, the deviance information criterion (DIC) statistic was calculated for each model to evaluate and used to compare model performance. A model with lower DIC was considered as one with a better fit.

We started with non-informative prior and 10,000 iterations. We checked for convergence of parameters visually using history plot and kerenel density estimate. Convergence was successfully achieved after 10,000 iterations for stunting and 20,000 iteration for severe stunting models. After convergence, a further 20,000 iterations were run and values were thinned by 10 and stored. The stored samples were used to calculate summary statistics (mean, SD, and 95% credible intervals) of the parameters. (Details of the structure of the models are described in S1 Dataset).

### Ethical clearance

The study protocol was approved by Institutional Review Board of Addis Ababa University, College of Health Sciences. The study was also approved by the Regional Committee for Medical and Research Ethics, Western Norway (REK Vest). Information on the research objective was read to the participants and verbal Informed consent was received. Verbal consent procedure was considered and approved according to the IRB/ethics committee approval. We obtained only verbal consent since the great majorities of the respondents were illiterate and could not write. This procedure is approved by Institutional Review Board of Addis Ababa University, College of Health Sciences as well as by the Regional Committee for Medical and Research Ethics, Western Norway (REK Vest). Privacy and confidentiality of respondents was also maintained.

## Results

The study involved a total of 4,094 children 0–59 months; 104(2.6%) of them had missing information on age, sex, or height or the values collected on age or height were not plausible. Fifteen (0.35%) households were not surveyed after repeated visits because of unavailability, which gives a final analytical sample size of 3975.

As a brief description of the background characteristics about the children included in the study, most of them lived with mother who had no formal education (75.3%); 15.2% were born in a health facility and nearly 93% of children lived in male headed households. More than 75% of the children lived in a household with a lowest dietary diversity and 81% lived in households with a moderate and mild food insecurity. Over three forth (77%) lived in a household with traditional pit latrine and 38% had morbidity in the past two weeks (see Table 1).

**Table 1:** Stunting according to demographic, household food security and other selected characteristics in 3975 children aged 0–59 months, Butajera, Ethiopia 2014.

Background characteristics	Height-for-age Z scores (HAZ)				
	Percentage withZ-score <—2	Percentage withZ-score <—3	Mean Z-Score	Weighted number ofchildren	Unweighted number of children
**Age in months**					
<6	19.3	14.1	-0.29	297	292
6–11	31.5	16.5	-0.98	408	408
12–23	51.7	27.5	-1.87	708	707
24–35	53.4	27.0	-2.11	742	731
36–47	45.4	20.7	-1.98	908	892
48–59	41.2	16.4	-1.88	971	945
P value (dose response trend)*	*P<0*.*01*	*P<0*.*05*			
**Sex of the child**					
Female	40.6	17.9	-1.64	2034	2006
Male	46.8	24.7	-1.83	2006	1969
**Place of delivery**					
Home	45.2	22.3	-1.78	3418	3370
Health facility	34.8	16.3	-1.49	615	598
**Mother marital status**					
Single, separated, divorced, widowed	42.0	21.9	-1.87	206	199
Married or living together	43.7	21.3	-1.73	3845	3776
**Mother’s Education**					
None	44.2	22.4	-1.78	3023	2965
Read and write	41.7	17.5	-1.67	151	149
Primary and above	41.5	18.1	-1.56	842	826
P value (dose response trend)*	*P<0*.*05*	*P<0*.*05*			
**Morbidity status (past 24 hr)**					
None	43.3	21.1	-1.78	2495	2463
Morbid	44.3	21.7	-1.67	1556	1530
P value (dose response trend)*					
**Household head gender**					
Female	38.8	21.5	-1.77	286	277
Male	44.0	21.4	-1.73	3722	3656
**Household food insecurity**					
Food secured	39.4	16.2	-1.55	772	745
Mildly food insecure	42.0	21.8	-1.74	1427	1403
Moderately food insecure	46.4	22.7	-1.82	1759	1739
severely food insecure	49.6	13.8	-1.49	59	55
P value (dose response trend)*					
**Household Food diversity score**					
Lowest	44.5	18.1	-1.77	637	619
Medium	43.8	22.0	-1.73	3189	3138
High	38.1	21.1	-1.69	225	218
**Household wealth**					
Poorest	47.6	24.3	-1.83	990	975
Poor	45.1	23.0	-1.82	747	728
Middle	43.2	21.1	-1.75	731	724
Rich	41.9	18.8	-1.66	900	879
Richest	39.0	18.7	-1.59	683	669
P value (dose response trend)*	*P<0*.*01*	*P<0*.*05*			
Household Dietary diversity score (0–12)**	4.12 ± 0.9	4.11± 0.9**	-	3975	3975
**Total [95%CI]**	43.7[40.9,46.4]	21.3[19.5,23.3]	-1.74	4048	3975

* Dose response test: The test performed is the Extended Mantel Haenszel Chi Square for linear trend with a p-value for one degree of freedom.

** mean and standard deviation

### Children’s nutritional status

A summary of the demographic, household food security and other selected characteristics of 3,975 children aged 0–59 months are presented in Table 1. The overall prevalence of stunting in the district was 43.7% [95%CI: 40.9, 46.4]. The prevalence of stunting started to rise at the age of 6–11 months (31.5%) and peaked at the age of 24–35 months (53.4%).The prevalence of stunting was found to be higher among children who were delivered at home (45.2%), whose mother were without any formal education (44.2%), and among those who lived in severely food insecure (49.6%) and poorest households (47.6%). The overall district level prevalence of severe stunting was 21.3% [95%CI: 19.5, 23.3]. Similar to the pattern observed for stunting, the prevalence of severe stunting started to increase at the age of 6–11 months (16.5%) and peaked at the age of 24–35 months (27.0%).

The result on the magnitude of food insecurity indicated that only 775 (19.2%) households were food secured while most of the sampled households were either mildly or moderately food insecured. A high percentage of households (78%) had a medium diversity score (consumed 4 or five food groups in the previous day) whereas 15.7% of the sampled households had a lowest dietary diversity (consumed 3 or less food groups in the previous day). The overall mean dietary diversity score was 4 food groups and did not vary among children.

A significant dose response linear relationship between stunting (severe stunting) and child age was documented (P<0.01). In addition to this, a significant dose response relationship was documented between stunting (severe stunting) and maternal education, household food insecurity level, and household wealth. Conversely, we did not observe noticeable differences in the prevalence of stunting (severe stunting) due to differences in marital status, gender of household head, and child’s morbidity status.

### Spatial distribution of stunting

The geographic distribution of stunting is shown in Fig 1. Using LISA statistics, we identified significant spatial variability on the distribution of stunting in the district. We identified statistically significant clusters of high prevalence of stunting (hotspots) in the eastern part of the district and clusters of low prevalence (cold spots) in the western (Fig 1).

**Fig 2:**
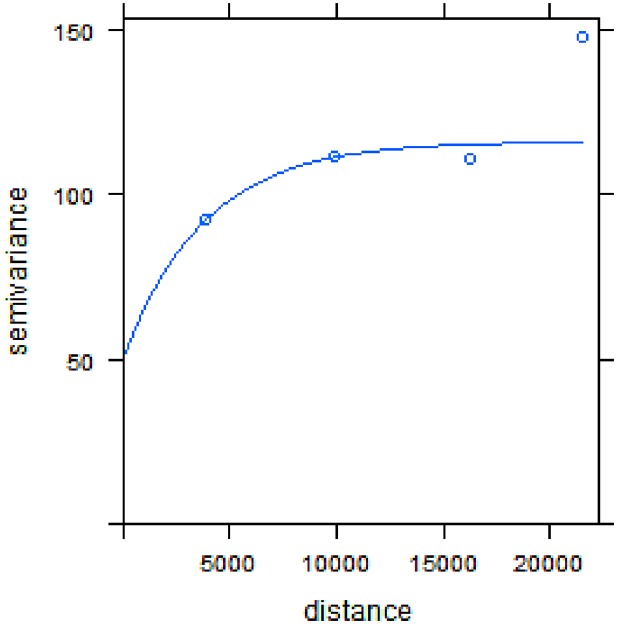
Empirical semivariogram model of the spatial dependency of stunting (parameter values fitted were range = 14400 meters, sill = 122.

Spatial autocorrelation was evident, as depicted in Fig 2. According to the semivariogram, stunting prevalence is spatially dependent at a maximum distance of 14,4 kms. However, our Bayesian multivariate models show that, after adjusting by individual and household-related covariates and accounting for random spatial effects, the range of spatial effect for stunting and severe stunting is around 33km. The geostatistical models for stunting showed that the range of spatial effects and spatial variances for stunting and severe were around 33km and 0.96 respectively (Table 2).

**Table 2:** Posterior means and standard deviation of regression coefficients (odds ratio and Bayesian credible interval) in the binary logistic geostatistical model for risk factor for childhood severe stunting Butajera, Ethiopia 2014.

Background characteristics	Severe Stunting (HAZ <−3)							
	Bayesian logistic regression model, no geostatistical component				Bayesian geostatistical model			
	Posterior Mean	SD	Median	Adjusted 95% Bayesian credible intervals (BCI) OR (95% BCI)	Posterior Mean	SD	Median	Adjusted 95% Bayesian credible intervals (BCI) OR (95% BCI)
***Age (months)***								
< 6	-	-		*1*.*0*	-	-	-	*1*.*0*
6–11	1.00	0.2	1.0	1.00 (0.60–1.60)	1.00	0.25	0.97	1.00 (0.61–1.56)
12–23	2.10	0.4	2.0	2.10 (1.40–3.00)*	2.05	0.43	2.00	2.05 (1.37–3.04)*
24–35	2.70	0.6	2.6	2.70 (1.80–4.00)*	2.67	0.56	2.61	2.67 (1.76–3.94)*
36–47	2.00	0.4	1.9	2.00 (1.30–2.90)*	1.96	0.41	1.92	1.96 (1.29–2.89)*
48–59	1.70	0.3	1.6	1.70 (1.10–2.50)*	1.66	0.35	1.62	1.66 (1.11–2.45)*
**Sex of the child**								
Female	-	-	-	1.0	-	-	-	1.0
Male	1.30	0.1	1.30	1.30 (1.10–1.60)*	1.34	0.11	1.34	1.34 (1.14–1.57)*
**Morbidity**								
None	-	-	1	1.0	-	-	-	1.0
Yes	0.9	0.1	0.9	0.90 (0.70–1.00)	0.86	0.07	0.86	0.86 (0.73–1.01)
**Place of delivery**								
Home	-	-	-	1.0	-	-	-	1.0
Health facility	0.90	0.1	0.90	0.90 (0.70–1.10)	0.91	0.11	0.90	0.91 (0.71–1.14)
**Mother’s Education**								
Primary and above	-	-	-	1.0	-	-	-	1.0
Read and write	0.90	0.2	0.90	0.90(0.50–1.50)	0.94	0.24	0.91	0.94(0.55–1.47)
None	1.30	0.1	1.30	1.30(1.10–1.60)*	1.33	0.14	1.33	1.30(1.08–1.64)*
**Ethnicity/race**								
Guraghee	-	-	-	1.0	-	-	-	1.0
Siltie	1.2	0.1	1.2	1.20(1.00–1.50)	1.25	0.14	1.25	1.25(1.00–1.54)
Mareko	1.4	0.3	1.4	1.40(0.96–2.00)	1.35	0.27	1.33	1.35(0.88–1.95)
Others	1.0	0.2	1.0	1.00(0.60–1.50)	0.97	0.22	0.95	0.97(0.59–1.45)
**Household food insecurity**								
Food secured	-	-	-	1.0	-	-	-	1.0
Mildly food insecured	1.3	0.2	1.3	1.30(1.00–1.60)	1.29	0.16	1.29	1.29(1.01–1.64)*
Moderately food insecured	1.3	0.2	1.3	1.30(1.00–1.60)	1.31	0.16	1.30	1.31(1.04–1.64)*
Severely food insecured	0.7	0.3	0.7	0.70(0.30–1.40)	0.71	0.31	0.66	0.71(0.26–1.45)
**Household wealth**								
Richest	-	-	-	1.0		-	-	1.0
Rich	0.8	0.1	0.8	0.80(0.60–1.00)	0.80	0.11	0.79	0.80(0.61–1.02)
Middle	0.9	0.1	0.9	0.90(0.70–1.20)	0.89	0.13	0.88	0.89(0.66–1.17)
Poor	1.0	0.1	1.0	1.00(0.80–1.40)	1.04	0.14	1.03	1.04(0.78–1.35)
Poorest	1.1	0.1	1.1	1.10(0.80–1.40)	1.10	0.14	1.09	1.10(0.84–1.41)
**attitude**								
altitude (meter)	1.0	0.0	1.0	1.00(1.00,1.00)	0.99	0.0	0.99	0.99(0.99,1.00)
**Model DIC**	**3890**				**3889**			
**Range of the spatial effect [range in km]**	**32.4 (6.2–202.5)**							
**Spatial variance**	**0.96(3.65E14–0.099)**							

### Potential risk factors

The Bayesian spatial and non-spatial models for stunting and severe stunting are presented in Tables 2 and 3. Based on the value of DIC statistics as a measure of model performance, the stunting model accounting for spatial random effects had substantially better model fit compared with the model without spatial component. However, the inclusion of spatial component in the severe stunning model did not improve the model fit.

**Table 3:** Posterior means and standard deviation of regression coefficients (odds ratio and Bayesian credible interval) in the binary logistic geostatistical model for risk factor for childhood stunting Butajera, Ethiopia 2014.

Background characteristics	stunting (HAZ <−2)							
	Bayesian logistic regression model, no geostatistical component				Bayesian geostatistical model			
	Posterior Mean	SD	Median	Adjusted 95% Bayesian credible intervals (BCI)OR (95% BCI)	Posterior Mean	SD	Median	Adjusted 95% Bayesian credible intervals (BCI) OR (95% BCI)
**Age (months)**								
< 6	-	-	-	1.0	-	-	-	1.0
6–11	1.70	0.30	1.60	1.70 (1.10,2.40)*	1.68	0.33	1.65	1.68 (1.14,2.42)*
12–23	4.10	0.70	4.00	4.10 (2.90,5.70)*	4.10	0.72	4.03	4.10 (2.90,5.72)*
24–35	4.70	0.80	4.70	4.70 (3.30,6.60)*	4.74	0.83	4.67	4.74 (3.35,6.58)*
36–47	4.00	0.70	3.90	4.00 (2.80,5.50)*	3.96	0.69	3.90	3.96 (2.82,5.50)*
48–59	3.20	0.60	3.10	3.20 (2.30,4.40)*	3.19	0.55	3.14	3.19 (2.28,4.41)*
**Sex of the child**								
Female	-	-	-	1.0	-	-	-	1.0
Male	1.3	0.1	1.3	1.3 (1.10,1.50)*	1.28	0.08	1.27	1.28 (1.12,1.45)*
**Place of delivery**								
Home	-	-	-	1.0	-	-	-	1.0
Health facility	0.8	0.1	0.8	0.8 (0.70–1.00)	0.8	0.08	0.81	0.81 (0.66–0.98)*
**Mother’s Education**								
Primary and above	-	-	-	1.0	-	-	-	1.0
Read and write	1.0	0.20	1.00	1.00(0.70,1.40)	1.02	0.20	1.00	1.02(0.68,1.45)
None	1.2	0.10	1.20	1.21(1.00,1.40)	1.21	0.10	1.20	1.21(1.02,1.42)*
**Ethnicity/race**								
Guraghee	-	-	-	1.0	-	-	-	1.0
Siltie	1.10	0.10	1.10	1.10(0.90,1.40)	1.14	0.11	1.13	1.14(0.94,1.36)
Mareko	1.30	0.20	1.30	1.30(0.90,1.80)	1.28	0.22	1.26	1.28(0.91,1.77)
Others	1.00	0.20	1.00	1.00(0.70,1.40)	0.97	0.18	0.95	0.97(0.66,1.37)
**Household food insecurity**								
Food secured	-	-	-	1.0	-	-	-	1.0
Mildly food insecured	1.10	0.10	1.10	1.10 (0.90,1.30)	1.08	0.10	1.07	1.08 (0.89,1.30)
Moderately food insecured	1.20	0.10	1.20	1.20(1.00,1.50)	1.26	0.12	1.25	1.26 (1.04,1.51)*
Severely food insecured	1.40	0.40	1.30	1.40(0.70,2.30)	1.36	0.42	1.31	1.36 (0.72,2.34)
**Household wealth**								
Richest	-	-	-	1.0	-	-	-	1.0
Rich	1.00	0.10	1.00	1.00 (0.80,1.20)	0.95	0.10	0.95	0.95 (0.76,1.18)
Middle	1.10	0.10	1.10	1.10 (0.90,1.40)	1.12	0.13	1.11	1.12 (0.89,1.40)
Poor	1.10	0.10	1.10	1.10 (0.90,1.40)	1.13	0.13	1.12	1.13(0.89,1.40)
Poorest	1.20	0.10	1.20	1.20 (1.00,1.50)	1.18	0.13	1.17	1.18(0.94,1.46)
**altitude (location)**								
altitude (meter)	1.0	0	1.00	1.00(1.00,1.00)	1.00	0	1.0	1.00(1.00,1.00)
**Model DIC**	**5133**				**5126**			
**Range of the spatial effect [range in km]**	**33 (6.5–204.5)**							
**Spatial variance**	**0.96(3.6E14–0.098)**							

The Bayesian geostatistical model for stunting indicated that age of a child, child’s sex, place of delivery, mothers education, and household food insecurity were significantly associated with stunting. Age and sex of a child, household food insecurity, and mother’s education were significantly associated with severe stunting.

We found that the risk of stunting and severe stunting increased as the age of the child increased. Children in the age group 24–35 months were 4.7 times higher odds of stunting (OR 4.74; 95% Bayesian credible interval [BCI]; 3.35–6.58) and 2.7 times more likely to be severely stunted (OR 2.67; 95% BCI; 1.76–3.94) relative to children below 6 months of age

The odds of stunting among boys was 28% higher than the odds among girls (OR 1.28; 95%BCI; 1.12–1.45) and the odds of severe stunting among boys was 34% higher than the odds among girls (OR 1.34; 95% BCI; 1.14–1.57).

Higher mother’s/guardian education level has a protective effect from the risk of stunting and severe stunting. The odds of stunting was 20% higher among children whose mother had no education compared to children whose mother had primary and above education (OR 1.21; 95%BCI; 1.02–1.42). Similarly, the odds of severe stunting was 30% higher among children whose mother had no education (OR 1.30; 95%BCI; 1.08–1.64). We also found that children born in a health facility were more likely to be stunted than children born at home (20% lower risk of stunting in children born home versus health facility, OR 0.81; 95%BCI: 0.66–0.986).

Household food insecurity is associated with increased risk of stunting and severe stunting. We found that children who lived in moderately food insecured households were more likely to be stunted than children living in food secured households. (OR 1.26; 95% BCI; 1.04–1.51).Moreover, children who lived in moderately food insecured households were more likely to be severely stunted than children living in food secured households (OR 1.31; 95% BCI; 1.04–1.64).

We didn’t find statistically significant association between the risk of stunting and risk factors such as ethnicity, household wealth, and altitude. Childhood morbidity, pace of delivery, household wealth, and place (location) of residency were not found to be associated with severe stunting.

## Discussions

In the south of Ethiopia, household food insecurity was identified as a major risk factor for stunting. Other factors such as child's age, sex, and place of delivery and mother's education also seem to contribute to the risk of stunting. We also found out a clear spatial pattern on the distribution of stunting at district level which would need further attention. This aggregation might be driven by environmental, anthropological and biological factors that have not been considered in this study. Additionally, our study also demonstrates the relevance of accounting for spatial dependency when exploring risk factors of stunting.

We found out that the inclusion of spatial structure of the data into the Bayesian model has shown to improve the model fit for stunting model. We also noted that some of the risk factors for stunting such as place of delivery, mothers’ education and household food insecurity become statistically significant when we included a random spatial effect in the model fitting. That means that other spatially dependent factors, which our model did not account for, are determining the risk and distribution of stunting in the study area. However we did not find an improvement in the model fit for severe stunting model when we included a spatial random effect.

In the spatial model, child characteristics such as child’s age and sex were important predictors for both stunting and severe stunting. Previous works in Ethiopia and elsewhere have shown that boys are more likely to be stunted and severely stunted than girls [27,40], even though this result is not consistent everywhere[41]. Further studies to understand the role of gender difference in the risk of stunting in the study setting and beyond is warranted.

The risk to stunting was greatest for the age group of 24–35 months. We found a significant dose response relationship between child’s age and the risk of stunting, indicating a possible cause-effect relationship. This relationship is also consistent with other similar works [41,42] and interpretable with the peculiarities specific to the age period [11]. This period (6–35 months) is the time when a child is introduced to complementary foods and poor complementary feeding practices during this age period is an important risk factor for stunting [43]. In addition, during this period children become more mobile and exposed to environmental factors influencing linear growth [11].

Mother’s education emerges as key predictor of stunting in this study. Mother’s education, which is a modifiable household factor, has a strong and consistent relationship with poor nutritional status [11,26–28]. We also documented a dose-response relationship between mothers/ caregiver’s education and stunting. Educational status of mothers/caregiver influences the risk to stunting in multiple pathways. Better maternal educational status probably improves child caring practices associated with stunting, or might help understand and respond to nutrition based educational messages, and eventually adopt optimal complementary feeding and household dietary behaviors [11].

Unsurprisingly, Household food insecurity (HFI) was found to be associated with stunting, as other works have also reported [20,44,45]. Children in moderately food-insecured households had 1.3 greater odds of being stunted than children in food-secured households. Similar to previous works, we noted that the relationship between HFI and stunting is independent of household wealth [20,44]. This might indicates that HFI is not simply an indicator of household socio economic status but can be an independent factor influencing stunting in children. This finding might suggest that, among resource constrained communities, interventions integrating HFI in nutrition programs would be effective in averting stunting [44]. It is also documented that HFI could impact stunting through other pathways than dietary diversity such as its effect on caregiving and feeding practices, and maternal depression [20].

We found non-significant relationship between household relative wealth and the risk of stunting. Some works have reported conflicting relations between household wealth (poverty) and stunting. Studies report that children living in a relatively wealthier household’s might be better off through improved access and consumption of goods and services which in turn improves undernutrition [20–22]. On the other hand, rising incomes at household’s level might not necessarily help to improve stunting. Vollmer and colleagues reported a weak relation between income growth and undernutrition at household level. The authors have indicated that this weak relationship could perhaps be due to the fact that the increased income might not be spent in a way that improves nutritional status of children [23–25]. Household spending on quality foods and resource allocation between food and non-food items could influence the success of the relative increase in income to avert child undernutrition at household level [24,25].

In summary, the risk factors identified in this study are in agreement with some studies and at same time differ from other reports. This might partly indicate the fact that the role of factors influencing the risk to stunting vary contextually. Furthermore, methodological differences may explain part of the differences in the findings of these studies. These differences could be related to differences on study design (i.e. some are cross sectional, others are cohort and ecological studies), sampling techniques, standardization of measurements and analytical methods employed (i.e. the use multilevel modeling, adjustment for clustering and spatial dependency, selection of exposure variables in the model).

Our study has potential limitations that require further consideration. The limitations stem from the methodology employed (study design and spatial analysis), measurements, and respondent behaviors. First, we could not ascertain child age with reports as there was no formal birth registration in the study community. Even though we developed a local events calendar for this purpose, we experienced age heaping during analysis. We were forced to categorize child’s and mother’s age for analysis. Second, we didn’t collect detailed dietary intake data (quantity and quality) for each children surveyed but for the entire family unit. The lack of a significant relationship between dietary diversity and stunting could be partly due to the fact that dietary diversity might not accurately reflect diet quality and micronutrient adequacy in the diet. Due to the target age for this analysis (0–59), we were not able to calculate and include indicators of breastfeeding and complementary feeding practices for children 0–24 months. The inclusion of these WHO IYCF indicators could have improved the model for stunting in our study. Third, due to the anticipation of support, women/respondent might have over reported deficiencies in dietary diversity which in turn had inflated the estimated level of food insecurity. Fourth, the statistical tests employed in the spatial analysis assumed stationarity and isotropy. This assumption hold that the population at risk is evenly distributed across the study area and the spatial dependency is the same in all directions. Finally, stunting and the potential risk factors were measured simultaneously using a cross sectional survey. However, stunting is a chronic condition and the result of a cumulative exposure of the child to various risk factors, whilst some of the risk factors measured in the study, such as dietary diversity and household food security, applied for a shorter period of time prior to the survey. Thus, we may not be certain that some of these risk factors measured in our study preceded the occurrence of stunting.

To mitigate some of the aforementioned limitations, we standardized the anthropometric measurements and applied a robust statistical analysis in a Bayesian framework. Furthermore, we accounted for the spatial dependence structure in the data for multivariate model fitting. On top of this, we tested the relationship between stunting and selected risk factors for a dose response relationship to take into account for possible cause -effect relationships.

## Conclusions

Stunting prevalence may vary across space at different scale. For this, it's important that nutrition studies and, more importantly, control interventions take into account this spatial heterogeneity in the distribution of nutritional deficits and their underlying associated factors. Thus, geographically targeted nutritional interventions might be more efficient and cost-effective in southern Ethiopia and, by extension, in similar settings. The findings of this study also indicated that interventions integrating household food insecurity in nutrition programs in the district might help to avert the burden of stunting.

## Supporting information

S1 Text Logistic geostatistical model structure and description. (DOCX)

S1 Dataset The data set used in the geostatistical model. (ZIP)
