# Supplementary material for: Spatial heterogeneity and risk factors for stunting among children under age five in Ethiopia: A Bayesian geo-statistical model
Source: PLoS One. 2017 Feb 7;12(2):e0170785. doi: 10.1371/journal.pone.0170785 (PMC5295674; doi:10.1371/journal.pone.0170785)
Supplement: S1 Text — (DOCX) [file pone.0170785.s001.docx]

**Supplemental Appendix**

Spatial heterogeneity and risk factors for malnutrition among children of fewer than five years: A Bayesian geostatistical model

**Binary logistic geostatistical model structure and description**

The binary logistic geostatistical models were of the form:

$$Y_{ij}\text{\textasciitilde}bernoulli\text{ (}p_{ij}\text{) ,}$$

$$logit (p_{ij})\text{ }= \alpha+ \sum_{i=1}^{p} \mathrm{\beta}_{i}\times\chi_{i}+\sum_{j=1}^{p} \mathrm{\beta}_{j}\times\chi_{i,j}+\nu_{i}+\theta_{i}$$

Where$Y_{ij}$is the status of malnutrition (stunted or severely stunted or not) of a child *i* in a village *j,* $p_{ij}$is the probability of a child *i* in a village *j* being stunted or severely stunted,$\alpha$ is the intercept, $\sum_{i=1}^{p} \mathrm{\beta}_{i}\times\chi_{i}$ is a vector of explanatory variables at child and household level multiplied by their coefficients$\mathrm{\beta}_{i}$,$\sum_{j=1}^{p} \mathrm{\beta}_{j}\times\chi_{i,j}$is a vector of explanatory variables at village level multiplied by their coefficients$\mathrm{\beta}_{j}$,$\nu_{i}$is the non-spatial error term (non-spatial random effect) and$\theta_{i}$is the residual spatial component (spatial random effect) which is defined by an exponential spatial correlation function.

We specified the model with non-informative prior for the intercept and the coefficients (mean equal to 0 and precision of 1x 10^6^). Three chains of models with an initial of 10000 iterations were run. The values of the coefficient and intercept were stored and evaluated for convergence using diagnostic tests. We checked for convergence visually using history plot and kerenel density estimate. Convergence was successfully achieved after 10000 iteration for stunting and after 20000 iteration for severe stunting. Once convergence was achieved, a further 20000 iterations was followed and the value were stored. The stored variables were also examined for the presence of autocorrelation. We observed significant autocorrelation for the intercept and the coefficient for latitude and it was decided to reduce autocorrelation by thinning subsequent sampling by10. The stored samples were used to calculate summary statistics (mean, SD, and 95% credible intervals) of the parameters
